# Gene expression analyses of GH/IGF axis in triploid crucian carp with growth heterosis

**DOI:** 10.3389/fendo.2024.1373623

**Published:** 2024-03-26

**Authors:** Weiling Qing, Bingxin Ren, Chenyi Lou, Huan Zhong, Yi Zhou, Shaojun Liu

**Affiliations:** State Key Laboratory of Developmental Biology of Freshwater Fish, College of Life Sciences, Hunan Normal University, Changsha, China

**Keywords:** triploid, growth, hybridization, polyploid breeding, nonadditive expression

## Abstract

Hybridization and polyploid breeding are the main approaches used to obtain new aquaculture varieties. Allotriploid crucian carp (3n) with rapid growth performance was generated by mating red crucian carp (RCC) with allotetraploids (4n). Fish growth is controlled by the growth hormone (GH)/insulin-like growth factor (IGF) axis. In the present study, we examined the expression characteristics of GH/IGF axis genes in hybrids F_1_, 4n, 3n, RCC and common carp (CC). The results showed that GHRa, GHRb, IGF1, IGF2, and IGF-1Ra were highly expressed in 3n compared with RCC and CC, whereas IGF3 was undetectable in the liver in RCC, CC and 3n. GHRa and GHRb had low expression in the 4n group. In hybrid F_1_, GHRa expression was low, whereas GHRb was highly expressed compared to the levels in RCC and CC. Moreover, in hybrid F_1_, the expression of IGF3 was higher, and the expression of IGF1 and IGF2 was lower than that in the RCC and CC, whereas the expression of IGF-1Ra was similar to that in RCC and CC. For the IGFBP genes, IGFBP1 had higher expression in 3n compared than that in RCC and CC, while other IGFBP genes were not high expressed in 3n. Among the genes detected in this study, 11 genes were nonadditively expressed in 3n, with 5 genes in the transgressive upregulation model. We proposed that the 11 nonadditive expression of GH/IGF axis genes is related to growth heterosis in 3n. This evidence provides new insights into hybridization and polyploid breeding from the perspective of hormone regulation.

## 1 Introduction

Hybridization and polyploid breeding are the most popular techniques in aquaculture and are always applied to generate advantageous traits, such as faster growth rate and stronger resistance (1–4). These two techniques are of great value in aquaculture and have produced significant economic benefits. Using these methods, an economically important aquatic fish allotriploid, allotriploid crucian carp (3n), which showed heterosis in growth, was produced (5, 6). First, the hybrid F_1_ was generated by mating red crucian carp (*Carassius auratus*, red var. ♀) (RCC) with common carp (*Cyprinus carpio*, ♂) (CC). Several fertile individuals were found in F_1_, which were suitable for producing subsequent generations, including F_2_ and F_3_, through self-mating. In F_3_, several individuals were allotetraploid (4n) (7). In addition, the allotetraploids were fertile. Subsequently, via multigeneration self-mating, the allotetraploids lasted for 30 generations. The allotetraploid lineage is a valuable resource for hybridization and polyploid breeding. Allotriploid crucian carp was generated by crossing the allotetraploid lineage (♂) with red crucian carp (♀) (8, 9). The allotriploid crucian carp was sterile and grew rapidly (10, 11). Because of these advantages, triploid fish have been widely introduced and farmed in China. To date, several studies have identified the mechanism underlying the faster growth rate of triploid fish (10, 12). These indicated that the allotriploid crucian carp, a sterile fish, may consume less energy during reproduction, accelerating body growth (13). However, the key molecular mechanisms of this heterosis still need to be fully understood, which may aid in developing the theory and application of polyploid breeding.

Fish growth, the focus of many researchers and breeders, is controlled by the growth hormone (GH)/insulin-like growth factor (IGF) axis (14, 15). GH is secreted by the pituitary gland and released into the circulatory system. GH plays functions by binding to its receptor (GHR) on the surface of target cells (16). The liver is the primary target tissue of GH. When GHR is activated, the liver produces IGF. Subsequently, the binding protein (IGFBP) binds to IGF and regulates the pathway by interacting with IGFR (17, 18). Two GHRs (GHRa and GHRb), three IGFs (IGF1, IGF2, and IGF3), one IGFR (IGF-1Ra), and eight IGFBPs (IGFBP1, IGFBP2a, IGFBP2b, IGFBP3, IGFBP5, IGFBP6a, IGFBP6b, and IGFBP7) have been identified in common carp and crucian carp. Owing to the polyploid evolution of these fish, the number of copies in their genome is large (19, 20). Thus, the number of genes related to the GH/IGF axis is high. The study of IGF system-related genes in polyploid fish not only provides a reference for gene expression in the background of fish polyploidy but also provides a theoretical basis for fish polyploid breeding. For example, previous study has shown that IGF3, a gene paralogous to other IGFs, has different functions in controlling gonadal development (21). This evidence provides new insights into gene evolution in fish during polyploidy events. Although we understand the basis of the GH/IGF axis in fish with different ploidies (12), many aspects of these functional genes still require further elucidation.

The allotriploid crucian carp has a growth advantage. It is also a suitable model for studying hybridization and polyploid breeding. Previous study has shown that the GH/IGF axis is related to faster growth in the allotriploid crucian carp (12). Additive and nonadditive expression are the two gene expression patterns in hybrids determined by compared to their parents. The additive expression pattern indicates that the gene expression level of the hybrid is equal to that of the mid-parent (average expression of parents), whereas the other expression models (the gene expression level of the hybrid is not equal to that of the mid-parent) are nonadditive. Several studies have suggested that nonadditive expression is associated with heterosis (22). Thus, it is important to investigate the expression of genes belonging to the GH/IGF axis in fish with different ploidy levels and to determine the relationship between gene expression profiles and growth rates. In the present study, the expression of key genes in the GH/IGF axis in red crucian carp, common carp, F_1_, allotetraploids, and allotriploid crucian carp were assayed. Gene expression models were assigned to additive/nonadditive expression models to show the association between gene expression characteristics and growth performance. These results provide a reference for distant hybridization and polyploid breeding.

## 2 Materials and methods

### 2.1 Experimental fish and materials

Common carp, red crucian carp, hybrid F_1_ allotetraploid, and allotriploid crucian carp were obtained from the State Key Laboratory of Developmental Biology of Freshwater Fish at Hunan Normal University (Changsha, China). These fish were cultured in five 20 m^2^ ponds. The daily feed amount was 2% of their body weight. All fish were 1-year-old and were collected in April 2021. For each type of fishes, 5 individuals were collected. MS-222 was used to anesthetize the fish before tissue collection, and the liver tissue was carefully harvested using scissors and placed in centrifuge tubes. Then, these tissues were frozen in liquid nitrogen and stored in a refrigerator at −80°C.

### 2.2 RNA extraction

Total RNA was extracted using RNAiso Plus (Trizol) reagent (Takara, Japan) according to the manufacturer’s instructions. The integrity of the extracted RNA was determined using 1.0% agarose gel electrophoresis, and the concentration and purity of the total RNA were determined using a NanoDrop-2000 spectrophotometer (Thermo Fisher Scientific Inc., MA, USA). Qualified samples were used to synthesize first-strand cDNA.

### 2.3 Synthesis of the first-strand cDNA

The first strand cDNA was synthesized using 1 μg total RNA per sample, using the PrimeScript™ RT Reagent Kit with gDNA eraser (Takara) according to the supplier’s instruction. After synthesis, the first strand cDNA was detected using reverse transcription PCR with a designed internal reference (using β-actin as the internal reference gene) (**Table 1**), and the qualified cDNA samples were detected by agarose gel electrophoresis. The cDNAs were used as templates for real-time quantitative PCR after a 40-fold dilution.

**Table 1:** Real-time quantitative PCR primers used in this study.

Target gene	Primer direction and sequence (5’–3’)	Product size (bp)
β-actin	forward -GCTCTTCCCCATGCAATCCT	180
	reverse -GGTTCCCATCTCCTGCTCAA	
GHRa	forward -CGACGACGTTGGTGTTGATTT	133
	reverse -TCGATGCCTTTTATTTTAGGTGC	
GHRb	forward-AACCTGGTCAAACAGCCTTTACTC	65
	reverse -AAAGCACAACTTCACCACATCG	
IGF-1Ra	forward -CCTCAAGAGCCTGCGATACAT	107
	reverse-GCTGACTCCAGTCCCACAGATAC	
IGF1	forward -TGCGTCCTCGCGTTGACT	130
	reverse -CATATCCTGTCGGTTTGCTGAA	
IGF2	forward -GAAAGGCTGCCCAGAGGTT	132
	reverse -TGTCGGGGAGGGTCATG	
IGF3	forward -GGCTTGTGTTTCTGAGGCAA	167
	reverse -TGTGTCAGTGGAAGGATGCTGT	
IGFBP2a	forward -GTGCCGTATGCCAAACAGAGC	191
	reverse -TCACCACGATGTCCATTCACT	
IGFBP2b	forward -AGAGGGGCGAGTGTTGGTG	129
	reverse -GCTGGCTGGTGGGTGGTT	
IGFBP3	forward -CCAGGCGTGCGGAGTGTA	177
	reverse -CCTTCCTCCTCAGGGTTATCGT	
IGFBP5	forward -GCGGTTGCTGCTTGACTTGC	179
	reverse -GGGTGGGTGCTGTGGTTTGTAT	
IGFBP6a	forward -GACCACATCCGTGTTGGCATT	130
	reverse -GACCCCTCGTCCCTGAAGC	
IGFBP6b	forward-AATAGCGGTGAAATAGAAAAGGC	121
	reverse- CACGAGTGTCACAGTTTGGGATG	
IGFBP7	forward -GAGGTTACTGGCTGGGTGC	144
	reverse-CTGGGAAGGAATGTCATTGATGG	

### 2.4 Real-time quantitative PCR

The primers of real-time quantitative PCR were designed using Primer Premier 5 (Premier Biosoft, USA). The specific sequences are listed in **Table 1**. The QuantStudio 3 Real-Time PCR System (ABI, USA) was used for quantitative PCR. The reagents used for real-time quantitative PCR were PowerTrack SYBR Green Master Mix (ABI), and the reaction volume was 10 μL, including 1 μL cDNA template, 0.5 μL forward primer, 0.5 μL reverse primer, 5 μL PowerTrack Green Master Mix, and 3 μL ddH_2_O. The prepared premix was added to a 96-well plate, which was placed on an instrument for amplification. Each reaction was repeated three times to ensure accuracy of the results. The PCR conditions were as follows: 50°C for 2 min, 95°C for 10 min, and 40 cycles of 95°C for 15 s, and 60°C for 45 s. The fluorescence was detected after the extension. A dissolution curve was used after the PCR reaction to detect the specificity of the PCR amplification. β-actin was used as the internal reference gene, and the relative quantitative values of the samples were calculated using the 2^-ΔΔCT^ method (23).

### 2.5 Statistics

All statistical analyses were performed using SPSS software (version 16.0; SPSS Inc., Chicago, IL, USA). The mean ± standard deviation was used to express the expression in each group. Differences in the components were examined using a one-way analysis of variance (ANOVA). The identification of gene expression patterns (additive/nonadditive expression) was based on previously published studies (24, 25). Additive expression is equal to half of the additive value of the gene expression in RCC and CC, whereas nonadditive expression is not equal to half of the additive value of the gene expression in RCC and CC. When the gene expression of hybrid had no difference from one parent while significant difference from the other parent, the gene was assigned to ELD pattern. When the gene expression of hybrid had significant high or low expression compared to both parents, the gene was assigned to transgressive upregulation or downregulation patterns, respectively.

## 3 Results

### 3.1 GHR gene expression characteristics

**Figure 1:**
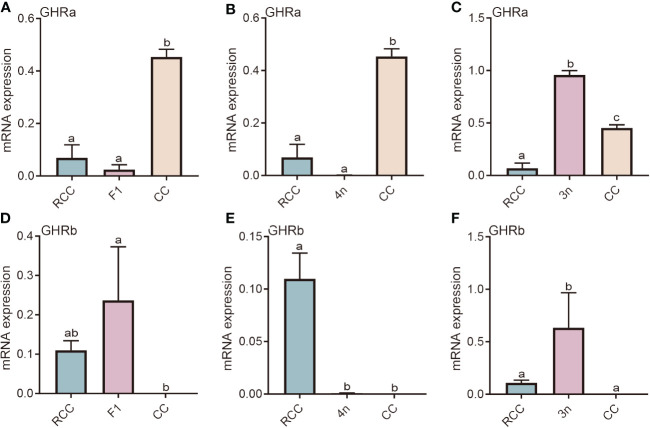
Expression analysis of GHR in liver of five fishes including red crucian carp, common carp, F_1_, allotetraploid crucian carp and allotriploid crucian carp. **(A)** Comparison of GHRa expression in RCC, F1 and CC; **(B)** Comparison of GHRa expression in RCC, 4n and CC; **(C)** Comparison of GHRa expression in RCC, 3n and CC; **(D)** Comparison of GHRb expression in RCC, F1 and CC; **(E)** Comparison of GHRb expression in RCC, 4n and CC; **(F)** Comparison of GHRb expression in RCC, 3n and CC. RCC stands for red crucian carp; CC stands for common carp; F_1_ stands for the first generation of red crucian carp (♂) × common carp (♀) hybrids; 4n stands for allotetraploid common carp; 3n stands for allotetraploid common carp. Groups with different lowercase letters in the column chart showed significant differences (*P*<0.05).

The liver is the major site of action of GH, and GHR is the key target protein. There are two types of GHRs in red crucian carp, common carp, hybridized F_1_, allotetraploids, and allotriploid crucian carp, including GHRa and GHRb. We compared the expression of hybridized F_1_ (F_1_), allotetraploid (4n), and allotriploid crucian carp (3n) with that of their original parents (RCC and CC). The GHRa expression levels in both RCC and F_1_ were significantly lower than those in CC (*P*<0.05) (**Figure 1A**). The gene expression of GHRa in 4n was similar to that in RCC, but both were significantly lower than that in CC (*P*<0.05) (**Figure 1B**), whereas the gene expression in 3n was significantly higher than that in RCC and CC (*P*<0.05) (**Figure 1C**). As for GHRb, the expression in F_1_ was not significantly different from that in RCC but was significantly higher than that in CC, whereas the expression in 4n was similar to that in CC but significantly lower than that in RCC (P<0.05) (**Figures 1D, E**). In 3n, GHRb expression was significantly higher than in RCC and CC (*P*<0.05) (**Figure 1F**).

### 3.2 Expression characteristics of IGF and IGF receptor

**Figure 2:**
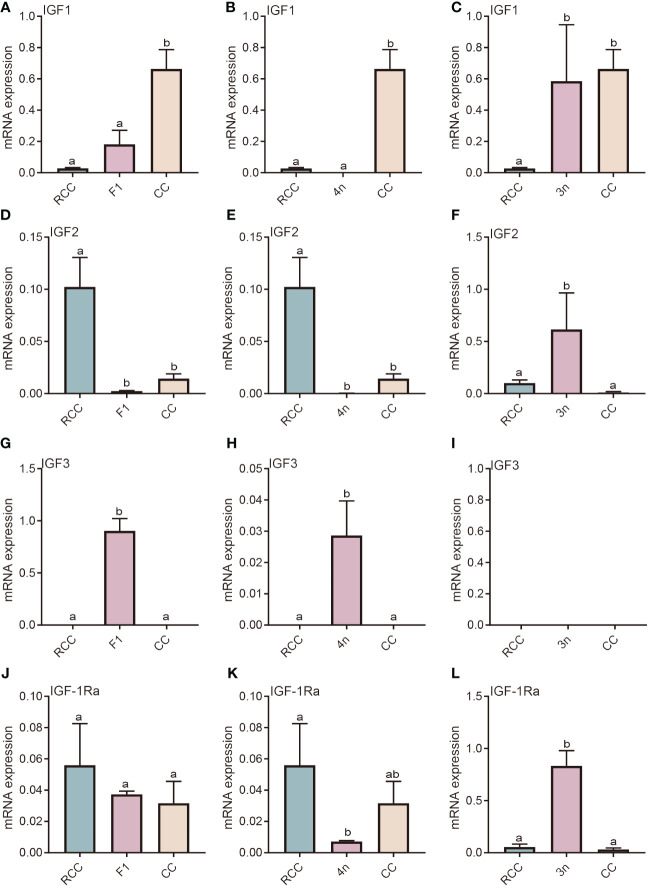
Expression analysis of IGF-related genes in liver of five fishes including red crucian carp, common carp, F_1_, allotetraploid crucian carp and allotriploid crucian carp. **(A)** Comparison of IGF1 expression in RCC, F1 and CC; **(B)** Comparison of IGF1 expression in RCC, 4n and CC; **(C)** Comparison of IGF1 expression in RCC, 3n and CC; **(D)** Comparison of IGF2 expression in RCC, F1 and CC; **(E)** Comparison of IGF2 expression in RCC, 4n and CC; **(F)** Comparison of IGF2 expression in RCC, 3n and CC; **(G)** Comparison of IGF3 expression in RCC, F1 and CC; **(H)** Comparison of IGF3 expression in RCC, 4n and CC; **(I)** Comparison of IGF3 expression in RCC, 3n and CC; **(J)** Comparison of IGF1-Ra expression in RCC, F1 and CC; **(K)** Comparison of IGF1-Ra expression in RCC, 4n and CC; **(L)** Comparison of IGF1-Ra expression in RCC, 3n and CC. RCC stands for red crucian carp; CC stands for common carp; F_1_ stands for the first generation of red crucian carp (♂) × common carp (♀) hybrids; 4n stands for allotetraploid common carp; 3n stands for allotetraploid common carp. Groups with different lowercase letters in the column chart showed significant differences (*P*<0.05).

The IGF-associated genes include IGF and its receptor (IGFR). There are three types of IGFs: IGF1, IGF2, and IGF3. IGF-1Ra is the main receptor of these IGFs. IGF1 gene expression in F_1_ and 4n was not significantly different from that in RCC but was significantly lower than that in CC (*P*<0.05) (**Figures 2A, B**). Significantly higher expression of IGF1 was found in 3n than in RCC (*P*<0.05) (**Figure 2C**). The expression patterns of IGF2 in F_1_ and 4n were similar, while IGF2 expression in the liver was significantly lower in the F_1_, 4n and CC than in RCC (*P*<0.05) (**Figures 2D, E**). IGF2 expression in 3n was significantly higher than that in RCC and CC (*P*<0.05) (**Figure 2F**). The expression of IGF3 in F_1_ and 4n was significantly higher than in RCC and CC, whereas the expression in 3n was undetectable (**Figures 2G–I**). The gene expression of IGF-1Ra in F_1_ was similar to that in RCC and CC (**Figure 2J**). The gene expression in 4n was similar to that in CC and significantly lower than that in RCC (*P*<0.05) (**Figure 2K**). The expression of IGF-1Ra in 3n was significantly higher than that in both RCC and CC (*P*<0.05) (**Figure 2L**).

### 3.3 IGFBP family gene expression characteristics

**Figure 3:**
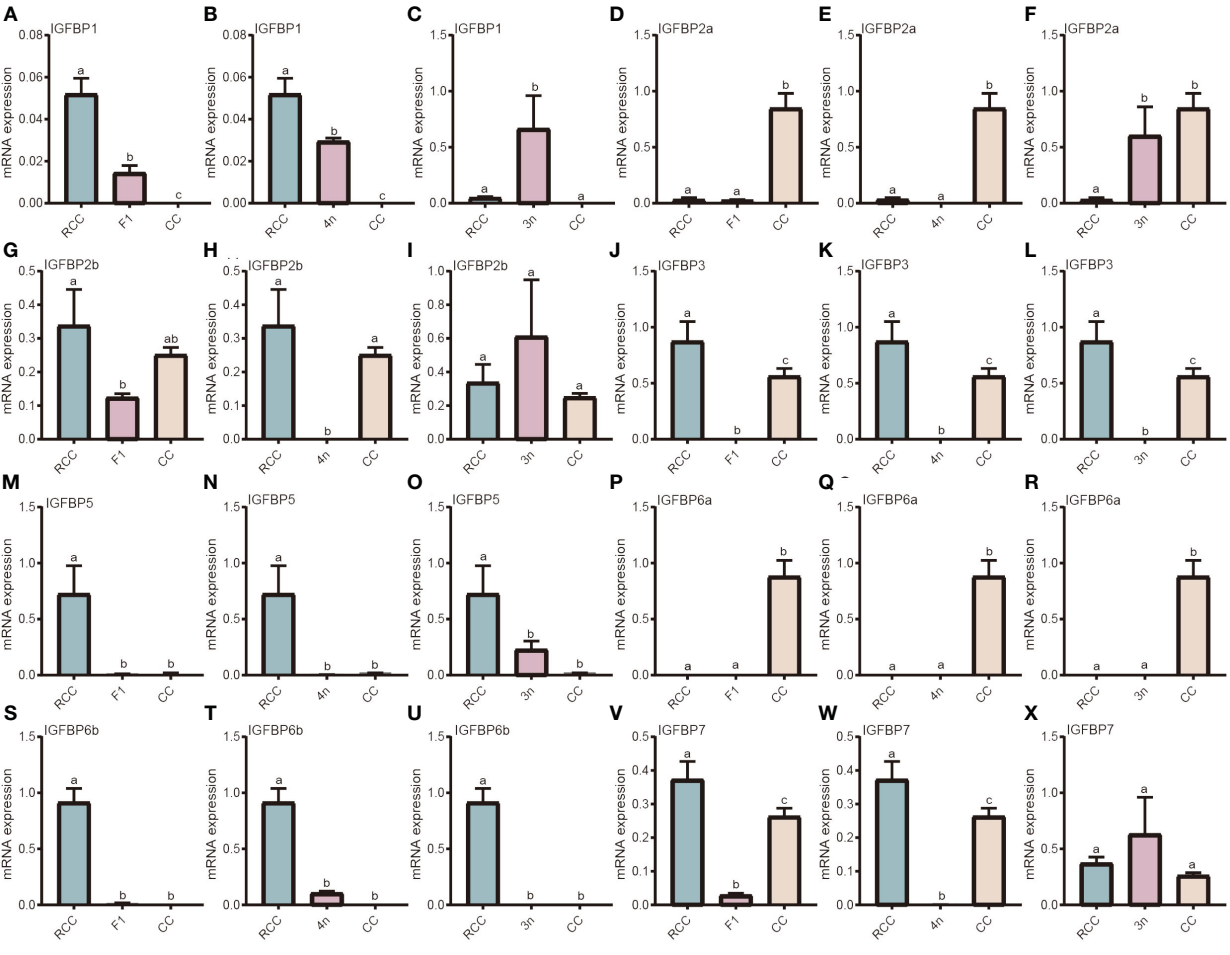
Expression analysis of IGFBP-related genes in liver of five fishes including red crucian carp, common carp, F_1_, allotetraploid crucian carp and allotriploid crucian carp. **(A)** Comparison of IGFBP1 expression in RCC, F1 and CC; **(B)** Comparison of IGFBP1 expression in RCC, 4n and CC; **(C)** Comparison of IGFBP1 expression in RCC, 3n and CC; **(D)** Comparison of IGFBP2a expression in RCC, F1 and CC; **(E)** Comparison of IGFBP2a expression in RCC, 4n and CC; **(F)** Comparison of IGFBP2a expression in RCC, 3n and CC; **(G)** Comparison of IGFBP2b expression in RCC, F1 and CC; **(H)** Comparison of IGFBP2b expression in RCC, 4n and CC; **(I)** Comparison of IGFBP2b expression in RCC, 3n and CC; **(J)** Comparison of IGFBP3 expression in RCC, F1 and CC; **(K)** Comparison of IGFBP3 expression in RCC, 4n and CC; **(L)** Comparison of IGFBP3 expression in RCC, 3n and CC; **(M)** Comparison of IGFBP5 expression in RCC, F1 and CC; **(N)** Comparison of IGFBP5 expression in RCC, 4n and CC; **(O)** Comparison of IGFBP5 expression in RCC, 3n and CC; **(P)** Comparison of IGFBP6a expression in RCC, F1 and CC; **(Q)** Comparison of IGFBP6a expression in RCC, 4n and CC; **(R)** Comparison of IGFBP6a expression in RCC, 3n and CC; **(S)** Comparison of IGFBP6b expression in RCC, F1 and CC; **(T)** Comparison of IGFBP6b expression in RCC, 4n and CC; **(U)** Comparison of IGFBP6b expression in RCC, 3n and CC; **(V)** Comparison of IGFBP7 expression in RCC, F1 and CC; **(W)** Comparison of IGFBP7 expression in RCC, 4n and CC; **(X)** Comparison of IGFBP7 expression in RCC, 3n and CC. RCC stands for red crucian carp; CC stands for common carp; F_1_ stands for the first generation of red crucian carp (♂) × common carp (♀) hybrids; 4n stands for allotetraploid common carp; 3n stands for allotetraploid common carp. Groups with different lowercase letters in the column chart showed significant differences (*P*<0.05).

Owing to the polyploid evolution of the fishes, the IGFBP family is large. IGFBP1, IGFBP2a, IGFBP2b, IGFBP3, IGFBP5, IGFBP6a, IGFBP6b, IGFBP7 were compared among the hybrids, RCC and CC. For IGFBP1, the gene expression of both F_1_ and 4n was lower than that in RCC but higher than that in CC (*P*<0.05) (**Figures 3A, B**), whereas the gene expression in 3n significantly surpassed that in RCC and CC (*P*<0.05) (**Figure 3C**). The gene expression of IGFBP2a in F_1_ and 4n was similar to that in RCC but significantly lower than that in CC (*P*<0.05) (**Figures 3D, E**), whereas the expression in 3n was similar to that in CC but higher than that in RCC (*P*<0.05) (**Figure 3F**). The gene expression of IGFBP2b was lower in F_1_ and 4n than in RCC and CC (*P*<0.05), whereas 3n showed no significant difference (**Figures 3G–I**). All hybrids (F_1_, 4n, and 3n) had lower gene expression of IGFBP3 than RCC and CC (*P*<0.05) (**Figures 3J–L**). The gene expression of IGFBP5 in F_1_, 4n and 3n was similar to that in CC but significantly lower than that in RCC (*P*<0.05) (**Figures 3M–O**). The expression patterns of IGFBP6a were similar in F_1_, 4n, and 3n, with lower expression in the hybrids and RCC than in CC (*P*<0.05) (**Figures 3P–R**). The expression of IGFBP6b in the hybrids and CC was significantly lower than that in RCC (*P*<0.05) (**Figures 3S–U**). The gene expression of IGFBP7 in F_1_ and 4n was lower than that in RCC and CC (*P*<0.05) (**Figures 3V, W**), whereas the gene expression in 3n was similar to that in RCC and CC (*P*>0.05) (**Figure 3X**).

### 3.4 Identification of nonadditive expression of GH/IGF axis genes in hybrids

**Figure 4:**
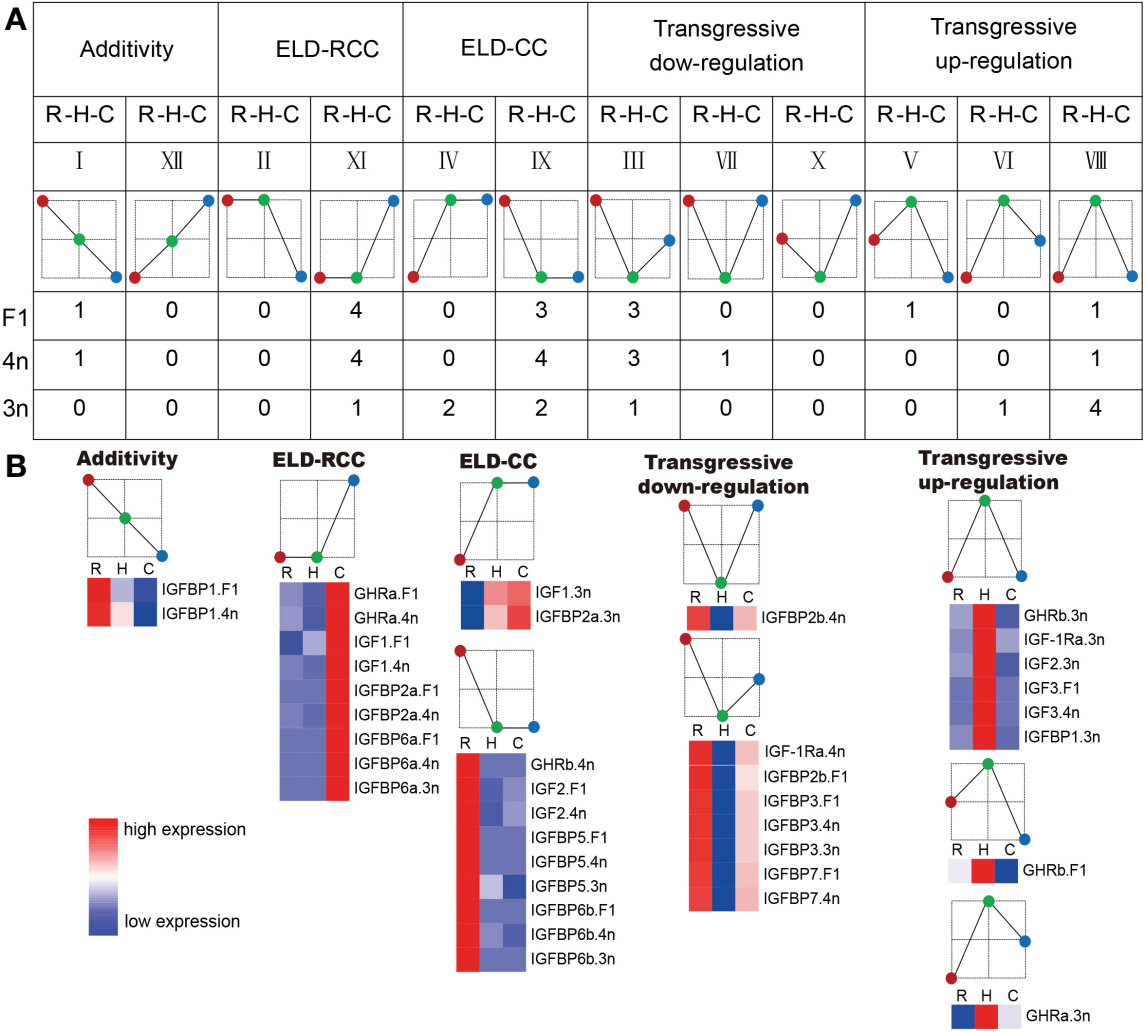
The gene expression pattern of F_1_, 4n, 3n compared to RCC and CC. **(A)** Assignment of the tested genes into the gene expression patterns. The number below the expression pattern diagram means the number of genes which has this expression pattern in F_1_, 4n, 3n, respectively. ELD-RCC means that the gene expression in hybrid is similar to that of RCC. Transgressive down-regulation means that the gene expression in hybrid is lower than both RCC and CC. The red circle means RCC. The green circle means hybrids. The blue circle means CC. R stands for red crucian carp. H stands for hybrid (F_1_, 4n, 3n). C stands for common carp. **(B)** Heatmap of the genes assigned to the expression patterns. In the heatmap, the color closer to red indicated that the gene expression was higher, and the color closer to blue indicated that the gene expression was lower.

Differentially expressed genes in the hybrids were assigned additive or nonadditive expression patterns. Nonadditive expression was found in the majority of GH/IGF axis genes. In F_1_, 12 genes were nonadditive, and 10 genes had ELD and transgressive downregulation patterns. Similar to F_1_, the majority of genes with nonadditive expression in 4n were identified with ELD and transgressive downregulation patterns (12 of 13 genes with nonadditive expression). In contrast, most genes with nonadditive expression in 3n showed a transgressive upregulation pattern, with five genes (45.45% of all the genes) showing nonadditive expression patterns (**Figure 4**).

## 4 Discussion

Previous research has shown that the advantages in the growth of allotriploid crucian carp (3n) are related to the high expression of the GH/IGF axis (12). Our study determined the expression pattern of genes in the GH/IGF axis from F_1_, 4n and 3n by comparing with their original parents to elucidate the molecular mechanism of the growth heterosis in 3n.

According to the present results, several genes in the GH/IGF axis were highly expressed in 3n. Compared with the RCC and CC, these gene expression patterns were predominantly nonadditive, which is consistent with rapid growth. A previous study showed that the GH/IGF system regulates the growth of bony fish (26). A previous study also found that in 3n, higher expression of GH, GHR, and IGF1 was found compared to that in RCC and 4n, both in the breeding and non-breeding seasons, which was in accordance with the fast growth appearance (12). However, gene expression was compared among the different ploidy fishes rather than among the hybrids and their parents in that study. This study focused on the nonadditive expression of these genes. In addition, the previous study only assayed GH, GHR, and IGF1; therefore, we analyzed the GHR, IGF, and IGFBP gene families, which covered more gene profiles in the GH/IGF axis. The results showed that almost all GHR and IGF genes (except IGF3) were significantly upregulated in 3n than in RCC and CC, indicating a nonadditive expression pattern. On the one hand, 3n showed faster growth performance, which may be due to the higher expression of GHR and IGF genes. In contrast, triploidization contributed to the excessive expression in 3n. Intriguingly, F_1_ and 4n may not have such coincidental expression patterns in 3n. Polyploidization has been used in both plants and animals. However, common rules for the occurrence of advanced phenotypes are yet to be elucidated. We hypothesized that these changes in gene expression can be attributed to epigenetic or genetic variations.

In the GH/IGF axis, the main functions of IGFBPs are to transport IGF, regulate the interaction of IGF with its receptor, and extend the half-life of IGF (27–29). Previous studies have shown that the IGFBP family is produced by replicating an ancestral IGFBP gene pair, and each IGFBP subunit has evolved its own function and structure (30). In this study, eight IGFBPs in the livers of five fish types were investigated. IGFBP2a and IGFBP2b are homologs of IGFBP2 in humans (31). Although their structures and functions are similar, their gene expression patterns have differed during evolution, as do those of IGFBP6a and IGFBP6b (32). It has been confirmed that all IGFBPs can inhibit IGF due to the high affinity between IGFBP and IGF (17). Although IGFBP6 inhibits IGF actions, it has been found that IGFBP1, IGFBP2, IGFBP3, and IGFBP5 can stimulate the actions of IGF, depending on the types of cells (28). In addition, the main effect of IGFBP7 is anticancer activity; however, some studies have found that it can also promote proliferation and differentiation (33). In mice, growth inhibition due to the high expression of IGFBP1, IGFBP2, IGFBP3, and IGFBP5 has been observed (17). Moreover, IGFBP1 and IGFBP2 have functions independent of IGFs, such as maintaining metabolic homeostasis (34). In the present study, IGFBP1 and IGFBP2a were highly expressed in 3n, whereas the expression of IGFBP2b and IGFBP7 was similar in RCC and CC. In contrast, IGFBP5 was expressed at low levels in 3n, whereas IGFBP3, IGFBP6a, and IGFBP6b were extremely low or undetectable. Most IGFBPs inhibiting growth were expressed at low or undetectable levels, similar to those associated with rapid growth. However, the abnormally high expression of IGFBP1 and IGFBP2 was the opposite. This may be related to maintaining metabolic homeostasis, which requires further investigation. In contrast, all IGFBPs had either low expression levels or were undetectable in F_1_ or 4n. This may be related to the low expression of IGFs. In 3n, except for IGFBP2b and IGFBP7, all others were expressed nonadditively, and this nonadditive expression may be caused by mutations and epigenetic modifications of drastic changes in the genome resulting from hybridization between 4n and RCC.

**Figure 5:**
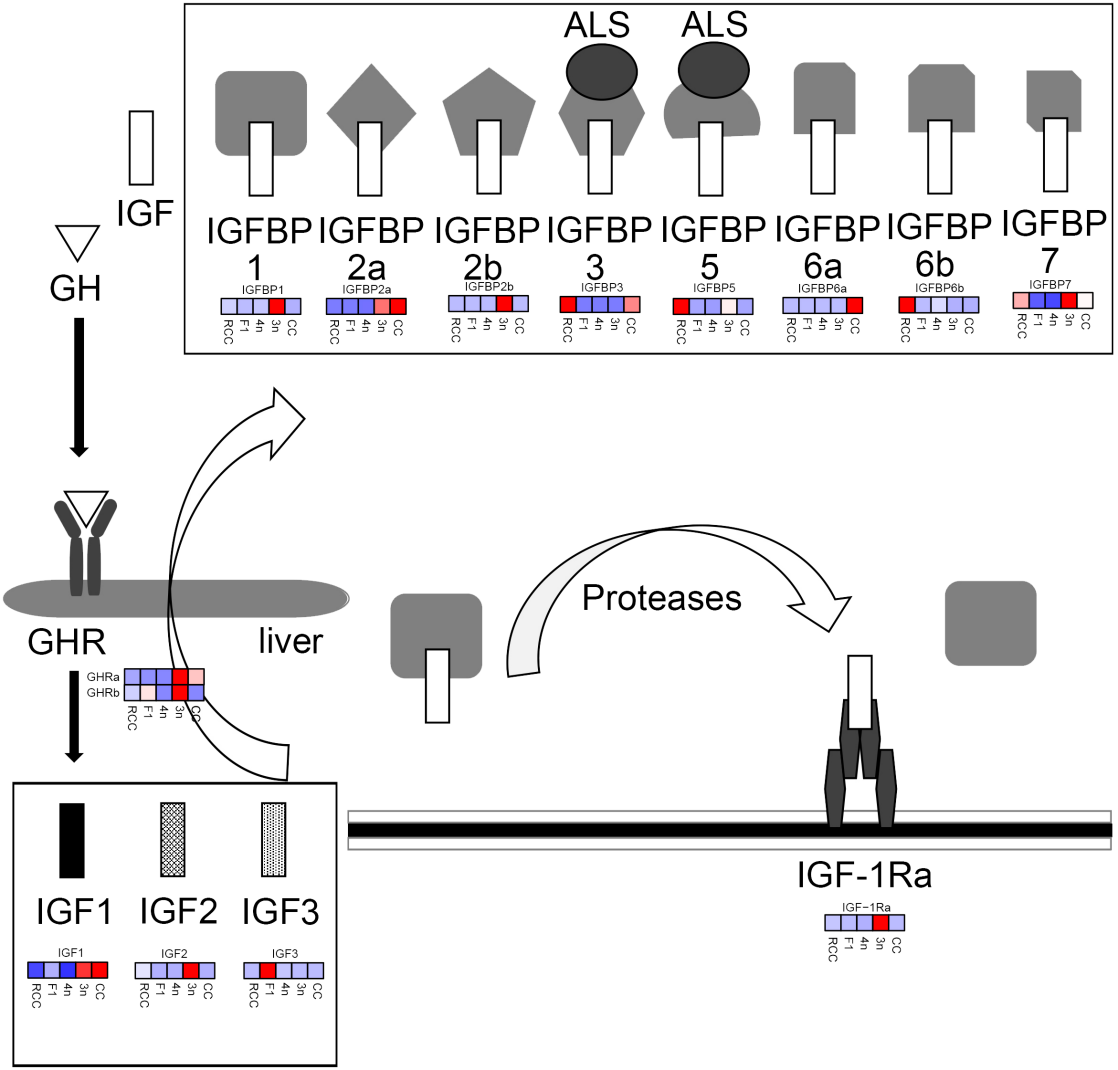
The expression profiles of GH/IGF axis genes. In heatmap, the color closer to red indicated that the gene expression was higher, and the color closer to blue indicated that the gene expression was lower. RCC stands for red crucian carp; CC stands for common carp; F_1_ stands for the first generation of red crucian carp (♂) × common carp (♀) hybrids; 4n stands for allotetraploid common carp; 3n stands for allotetraploid common carp.

Heterosis, which results from hybridization between different lineages or even species, has been considered a mechanism that could be utilized for breeding (35). In hybrids, the genomes of different species are mixed and interact, leading to genomic shock under stress and regulatory interference (36). Under these conditions, transposable elements may be mobilized, and gene expression can change dramatically, contributing to the advancement of phenotypes in hybrids (37, 38). Nonadditive gene expression is the reason for heterosis at the transcriptional level, which results from genomic shock (22). The present results showed that several GH/IGF axis genes were nonadditive in 3n, especially with a transgressive upregulation pattern showing the molecular mechanism for faster growth performance in 3n. The genes assigned to transgressive upregulation pattern in 3n included GHRa, GHRb, IGF-1Ra, IGF2 and IGFBP1. These genes contained GH receptors, IGF receptor, IGF2 and IGFBP1. Combining with previous reported results (12), these evidences suggested that high expression of GH/IGF axis genes in 3n compared to RCC and CC which is associated with its faster growth appearance. Compared with previous studies, the present study scanned the global expression of GH/IGF axis genes in 3n and its relatives, providing unprecedented details about the transcriptional landscape in different ploidy fishes (**Figure 5**). However, further studies are required to elucidate the transcriptional regulation of these functional genes.

In this study, the correlation of the GH/IGF axis with heterosis of allotriploid crucian carp was studied, and the related mechanism was accurate at the gene level, which can provide a reference for future research. However, the underlying mechanism remains unclear, and further experimental studies are needed.

## Data availability statement

The original contributions presented in the study are included in the article/supplementary material. Further inquiries can be directed to the corresponding authors.

## Ethics statement

The animal study was approved by The Animal Ethics Experimental Committee of Hunan Normal University, China. The study was conducted in accordance with the local legislation and institutional requirements.

## Author contributions

WQ: Data curation, Formal analysis, Investigation, Methodology, Resources, Validation, Visualization, Writing – original draft, Writing – review & editing. BR: Data curation, Formal analysis, Investigation, Methodology, Resources, Writing – original draft, Writing – review & editing. CL: Data curation, Formal analysis, Investigation, Methodology, Writing – review & editing. HZ: Data curation, Formal analysis, Resources, Software, Visualization, Writing – original draft, Writing – review & editing. YZ: Conceptualization, Funding acquisition, Investigation, Project administration, Supervision, Validation, Visualization, Writing – original draft, Writing – review & editing. SL: Funding acquisition, Project administration, Supervision, Validation, Writing – review & editing.
